# Case Report: An incidentally discovered HPV-associated endocervical adenocarcinoma presenting as pseudomyxoma peritonei

**DOI:** 10.3389/fonc.2025.1630879

**Published:** 2026-01-14

**Authors:** Lijun Chen, Lin Sun

**Affiliations:** 1 Department of clinical medicine, Jining Medical University, Jining, Shandong, China; 2 Department of Obstetrics and Gynecology, Affiliated Hospital Of Jining Medical University, Jining, Shandong, China

**Keywords:** adenocarcinoma, carcinoma cervix, metastasis, ovary, pseudomyxoma peritonei(PMP)

## Abstract

This case report describes a 48-year-old woman with occult HPV-associated endocervical adenocarcinoma (Silva pattern B) presenting as bilateral ovarian metastases and pseudomyxoma peritonei–like gelatinous ascites, despite normal cervical morphology and resolved HPV type 45 infection. Initial misdiagnosis as primary ovarian mucinous carcinoma was revised based on histopathology and immunohistochemistry (diffuse p16 positivity, ER-negative and PR-negative, Ki-67 index of 95%), confirming metastatic endocervical adenocarcinoma. The absence of appendiceal lesions excluded true PMP, attributing ascites to tumor mucin secretion. This case highlights the diagnostic challenges of occult cervical adenocarcinoma mimicking PMP and underscores the critical role of immunohistochemical profiling (p16, PAX8, WT1) to differentiate metastatic from primary ovarian tumors. The study emphasizes a multidisciplinary approach for accurate classification of mucinous neoplasms and raises awareness of rare metastatic pathways, such as transtubal dissemination, in HPV-associated cervical adenocarcinoma.

## 1 Introduction

Cervical cancer is the fourth most common cancer in women, with approximately 604,000 new cases reported in 2020 (1). Despite the decreased incidence of cervical squamous intraepithelial lesions and squamous cell carcinoma attributable to expanded screening, the relative frequency of endocervical adenocarcinoma has increased from 5% to 20% (2). The WHO Classification of Tumors of the Female Reproductive Organs (2014) primarily classified endocervical adenocarcinoma based on morphological features (3). The 2018 International Endocervical Adenocarcinoma Criteria and Classification (IECC) system proposed categorizing cervical adenocarcinomas by etiology into human papillomavirus (HPV)-associated adenocarcinoma (HPVA) and non-HPV-associated adenocarcinoma (NHPVA). This classification was subsequently adopted by the WHO Classification of Tumors of the Female Genital Tract (2020) (4). HPVA includes usual-type (villoglandular variant) and mucinous carcinoma (intestinal variant, signet-ring cell variant), while NHPVA comprises gastric-type adenocarcinoma, clear cell carcinoma, and other rare subtypes.

Approximately 90% of cervical adenocarcinomas are human papillomavirus (HPV)-associated cancers, with HPV16 and HPV18 being the most prevalent genotypes (5). These tumors are characterized by diffuse strong p16^INK4a^ overexpression and are typically negative for hormone receptors (estrogen receptor [ER]/progesterone receptor [PR]) (6). They typically disseminate through local invasion and lymphatic metastasis, whereas ovarian metastases are uncommon, and the underlying mechanisms remain unclear.

Clinically, cervical adenocarcinoma may manifest solely as ovarian space-occupying lesions while showing no obvious cervical abnormalities. This is particularly critical because diagnosing primary cervical adenocarcinoma remains challenging in asymptomatic women with normal cervical morphology and negative Papanicolaou (Pap) test results (6).Metastatic lesions of cervical adenocarcinoma typically manifest as pelvic masses and ascites, with the ascitic fluid usually being serous or hemorrhagic; gelatinous ascites is relatively uncommon. Pseudomyxoma peritonei (PMP) is a rare condition characterized by the implantation of mucus-secreting cells on the peritoneal surface, omentum, or visceral organs, leading to the accumulation of massive, yellowish, gelatinous mucinous ascites in the abdominal cavity. This report presents a case of occult HPV-associated cervical adenocarcinoma with bilateral ovarian metastases, in which the ascites resembled that seen in pseudomyxoma peritonei. This report aims to highlight this unusual presentation, thereby providing insights for the early diagnosis and management of cervical adenocarcinoma and serving as a diagnostic reference for similar cases.

## 2 Case report

**Figure 1:**
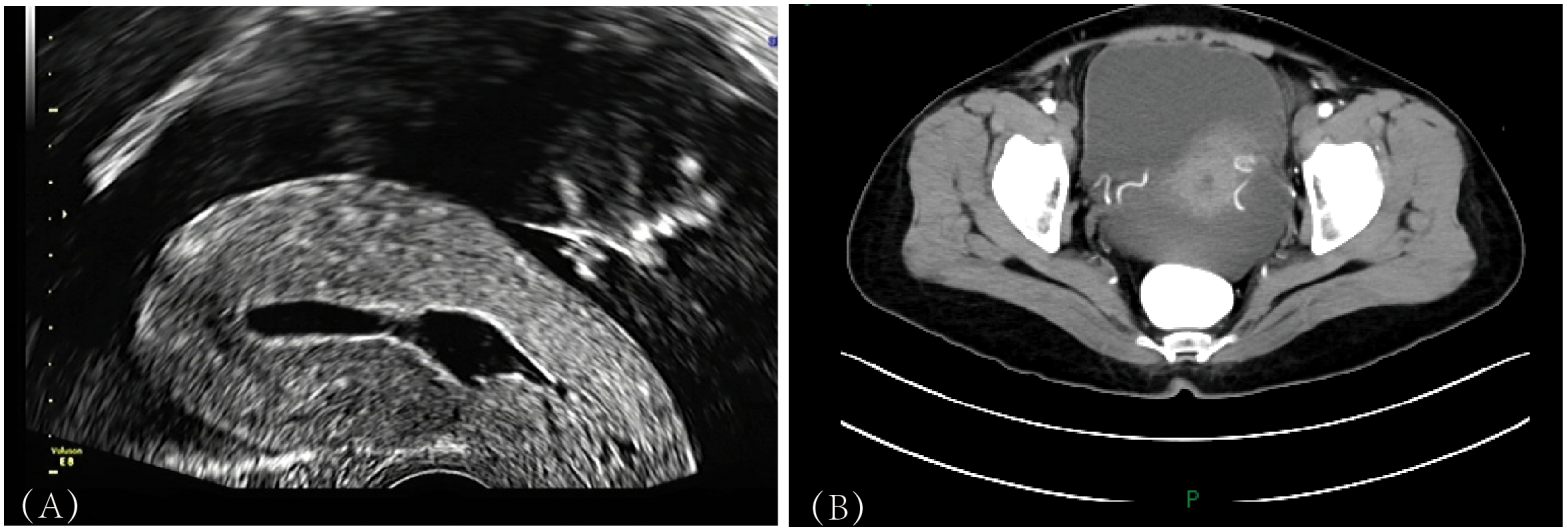
**(A)** Transvaginal ultrasound image showing a hyperechoic lesion within the endocervical canal. **(B)** Abdominal contrast-enhanced CT scan clearly demonstrating a large cystic-solid mass encasing both adnexa, accompanied by ascites.

A 48-year-old woman presented on February 10, 2025 with a one-month history of lower abdominal distension accompanied by tenderness and pain, along with heartburn and fatigue. She had experienced spontaneous menopause for 2 years without postmenopausal bleeding or abnormal vaginal discharge. Her symptoms showed no improvement with self-administered Traditional Chinese Medicine (TCM). Transvaginal and transabdominal ultrasonography revealed: a hyperechoic lesion in the endocervical canal (0.8 cm × 0.6 cm) with well-defined margins, heterogeneous echogenicity, and vascular flow, suggestive of endocervical polyp (**Figure 1A**); a right adnexal complex mass (15.9 cm × 5.6 cm × 14.4 cm) with ill-defined borders; a left adnexal heterogeneous mass (6.8 cm × 2.0 cm × 3.8 cm) with indistinct margins; and pelviperitoneal free fluid. Gynecological examination revealed normal vulvar development, a patent vagina, and a smooth cervix without contact bleeding. The uterus was anteverted and normalized. A firm, 18-cm cystic-solid mass was palpated in the lower abdomen, with no nodules detected in the rectouterine pouch (pouch of Douglas).Contrast-enhanced CT demonstrated a large complex cystic-solid mass (21.1 cm × 9.9 cm × 23.2 cm) encasing bilateral adnexa, radiologically suggestive of malignant mucinous adenocarcinoma of ovarian origin(**Figure 1B**).The laboratory tumor marker results showed a mildly elevated level of Alpha-fetoprotein (AFP), while the remaining markers were within normal ranges (**Table 1**). The patient has a history of infection with HPV type 45. In May 2024, HPV genotyping test was negative, and the ThinPrep cytology test (TCT) result indicated: Negative for Intraepithelial Lesion or Malignancy (NILM). In the preoperative evaluation of this case, the patient reported no breast-related symptoms or history, and breast ultrasonography revealed no abnormalities. The patient reported no history of appendiceal disease. Preoperative abdominal ultrasound and the contrast-enhanced CT scan did not indicate any evident lesions within the gastrointestinal or biliary tracts. The preliminary diagnoses included pelvic mass, ovarian neoplasm, endocervical polyp, and pelviperitoneal effusion.

**Table 1:** Serum tumor marker test results.

Tumor marker	Test result	Reference range	Unit
Alpha-fetoprotein (AFP)	14.72	0-8.78	ng/mL
Carcinoembryonic Antigen (CEA)	2.16	0-5	ng/mL
Carcinoembryonic Antigen125 (CA-125)	12.8	0-35	U/mL
Carcinoembryonic Antigen19-9 (CA19-9)	26.68	0-37	U/mL
Human Epididymis Protein 4(HE4)	44.3	Premenopausal :0-70 Postmenopausal :0-140	pmol/L

**Figure 2:**
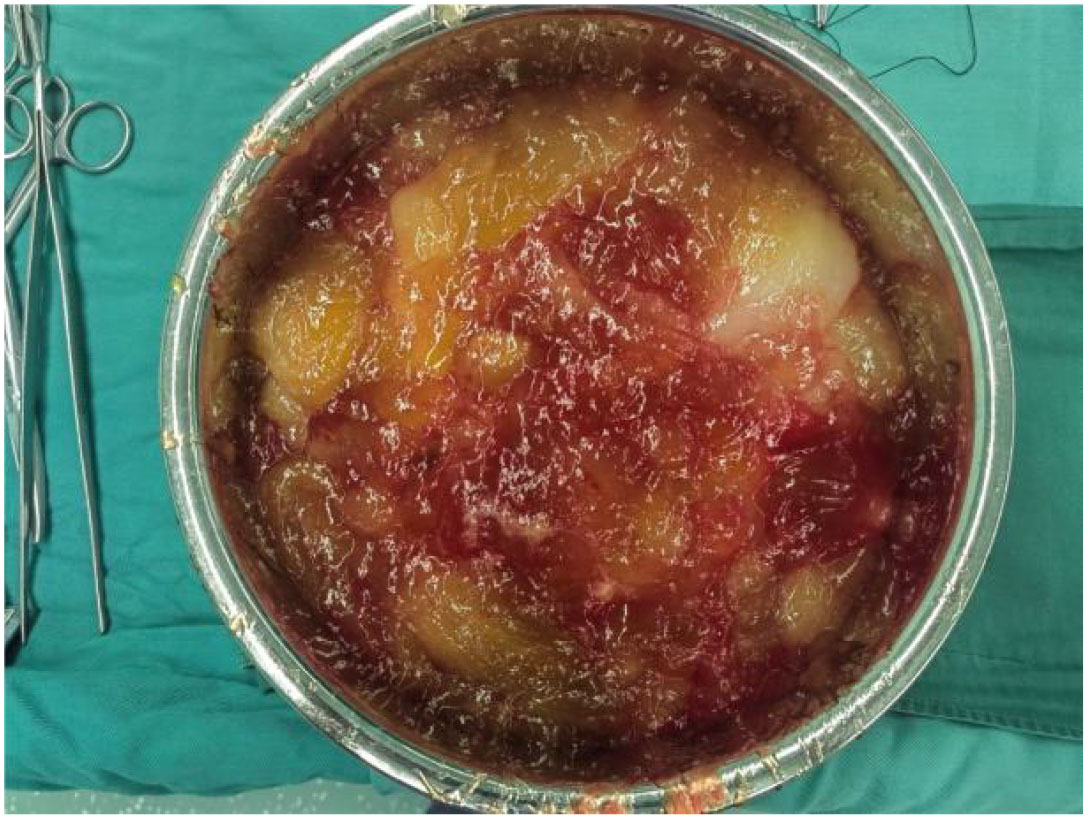
A large amount of straw-colored, gelatinous ascitic fluid was observed during the surgery.

**Figure 3:**
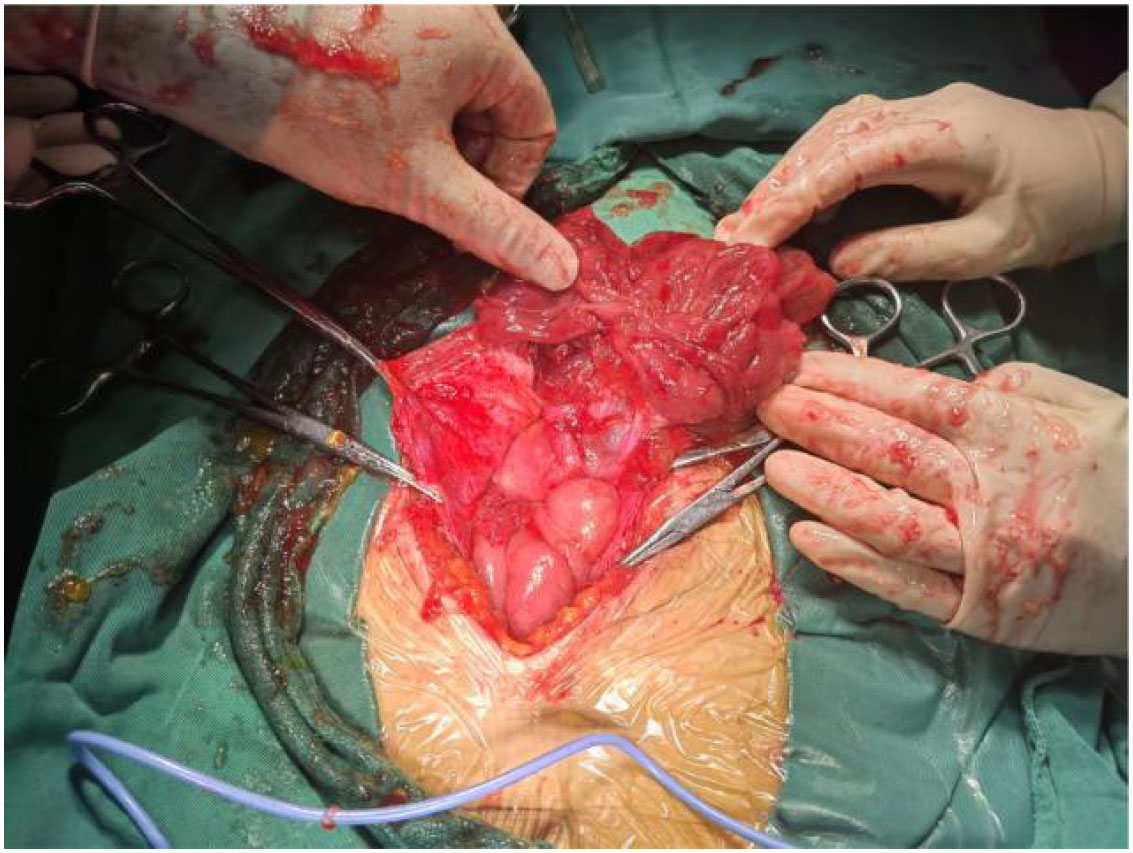
Right ovary: The gross specimen measured approximately 10 cm in diameter.

**Figure 4:**
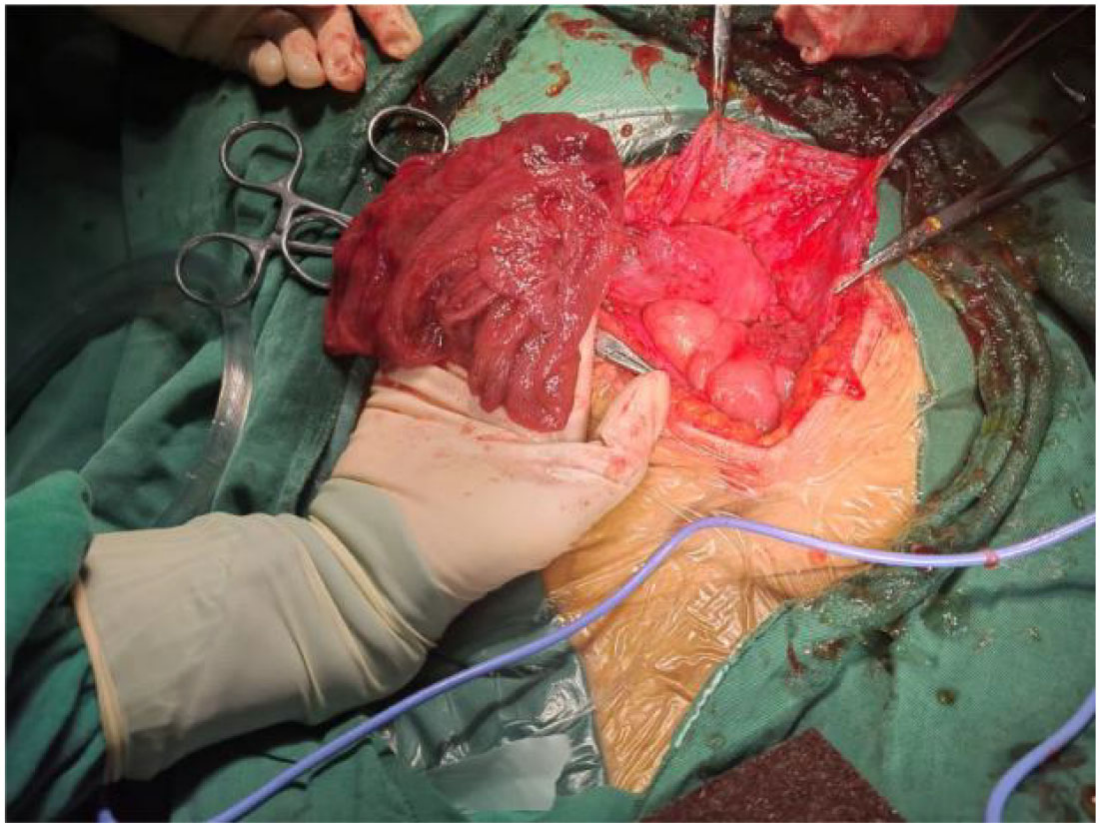
Left ovary: The gross specimen measured approximately 4 cm in diameter.

**Figure 5:**
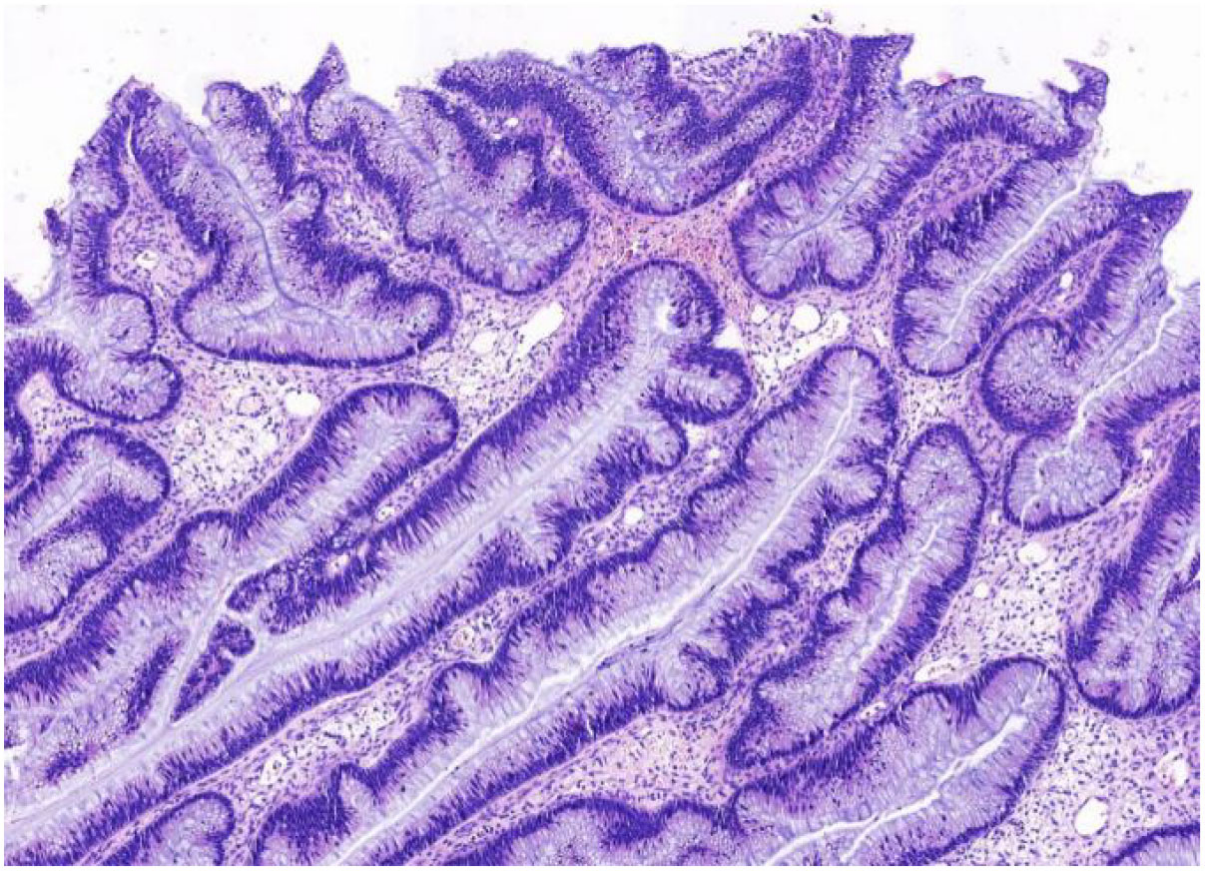
Intraoperative frozen section of the right ovary revealed a mucinous tumor, suggestive of a borderline lesion(H&E stain, ×100 ).

On February 12, 2025, an exploratory laparotomy was performed. Intraoperative findings included viscous amber-colored ascites (**Figure 2**). After ascites evacuation, the uterus appeared normal-sized, while both ovaries exhibited florid papillary excrescences with vivid red discoloration. The right ovary measured 10 cm in diameter (**Figure 3**), and the left 4 cm (**Figure 4**), with complete replacement of normal ovarian architecture. Both fallopian tubes showed no gross abnormalities. The right adnexa was resected for intraoperative frozen section analysis, which demonstrated a mucinous neoplasm with epithelial proliferation features consistent with a mucinous borderline tumor (atypical proliferative mucinous tumor). Definitive diagnosis awaited formalin-fixed paraffin-embedded (FFPE) histopathological examination (**Figure 5**).

**Figure 6:**
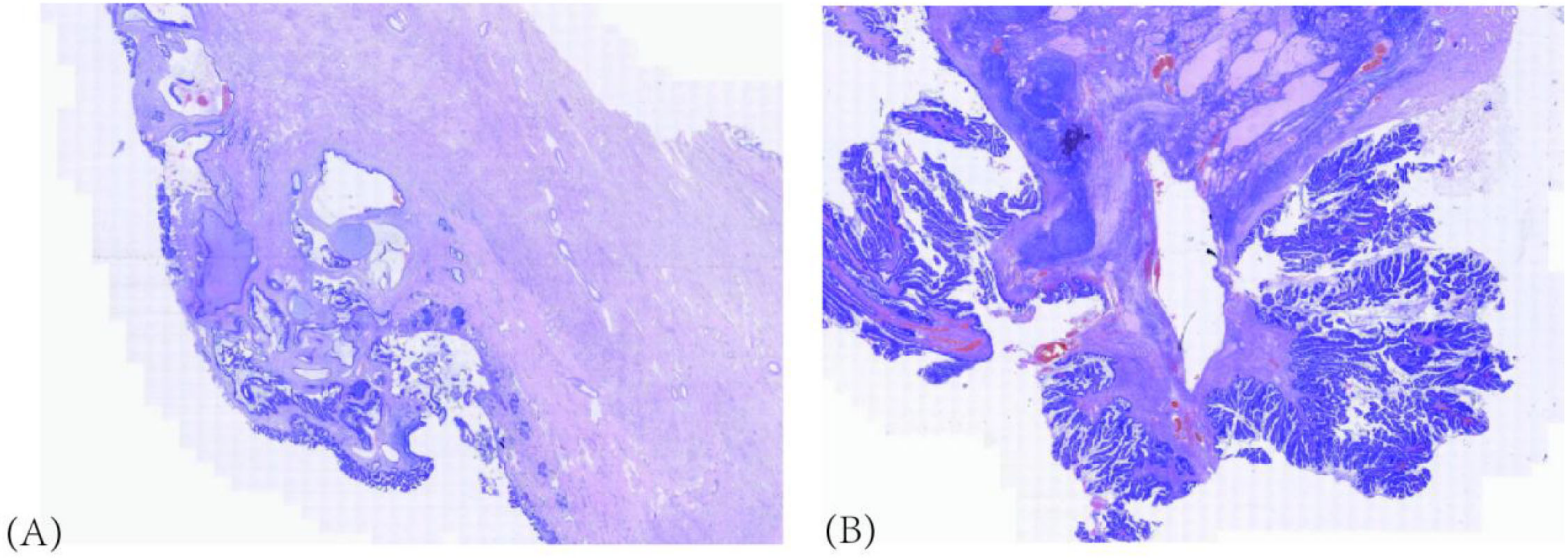
**(A)** Routine cervical pathology revealed HPV-associated endocervical adenocarcinoma, Silva pattern **(B)** The tumor involved nearly the entire cervical wall (approximately two-thirds) with extension to the endometrium. Numerous intravascular tumor emboli were identified. **(B)** Routine pathology of the left ovary indicated a mucinous tumor (H&E stain, ×50).

**Figure 7:**
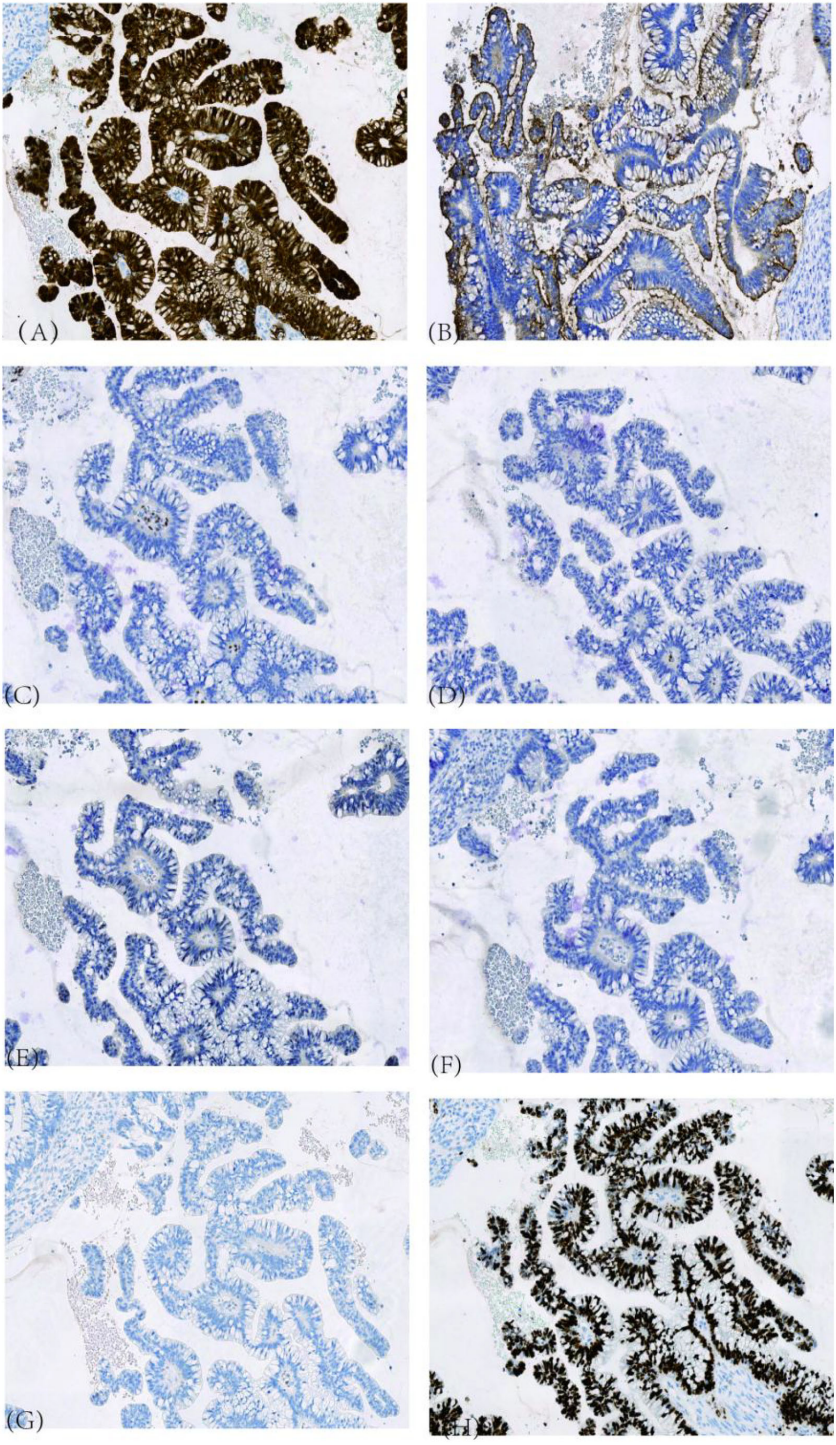
Immunohistochemical Results of Cervical Tissue (H&E stain, ×100). **(A)** P16, diffusely and strongly positive; **(B)** CEA, positive; **(C)** ER, negative; **(D)** PR, negative; **(E)** PAX-8, negative; **(F)** WT-1, negative; **(G)** p53, wild-type pattern; **(H)** Ki-67 index, approximately 95%.

**Figure 8:**
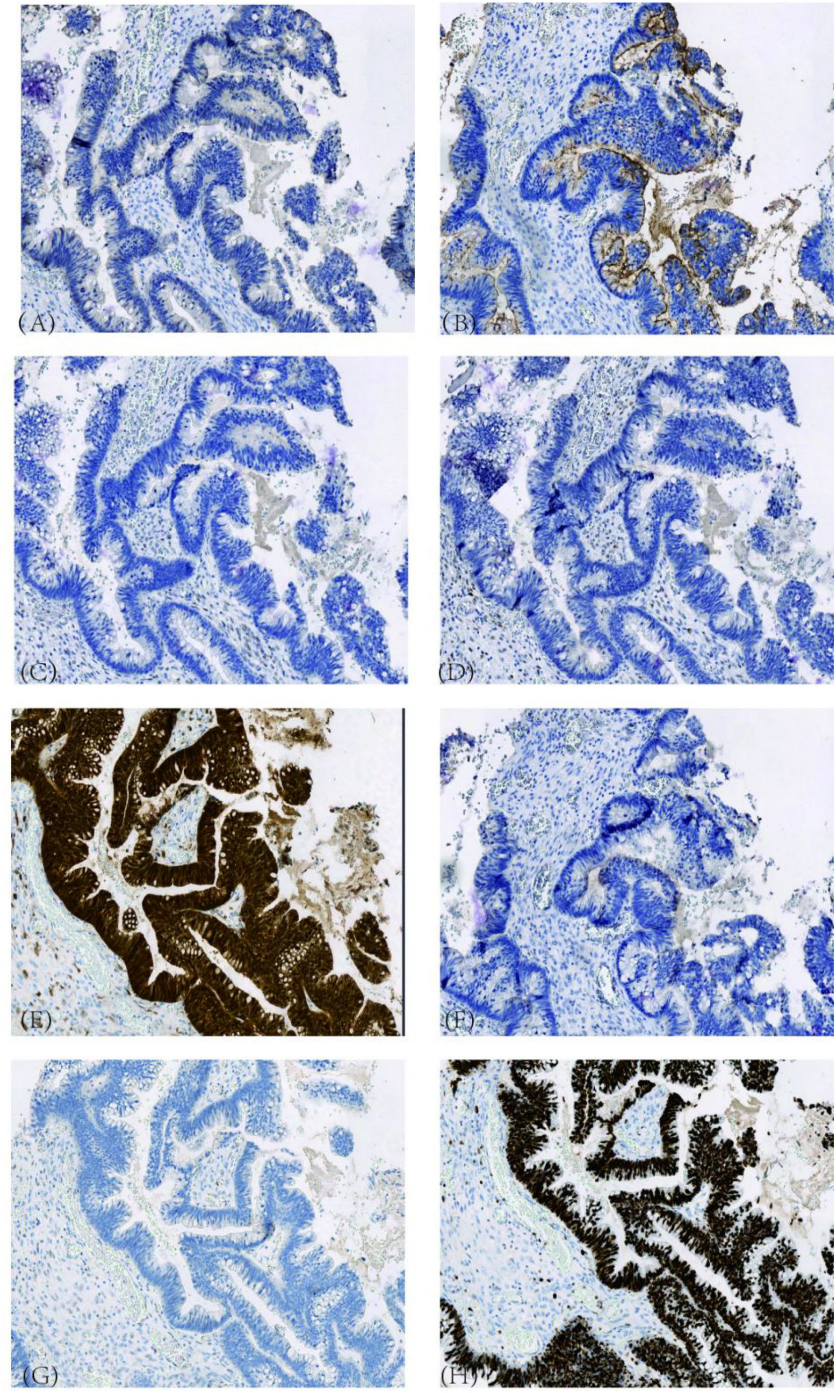
Immunohistochemical Results of Ovarian Tissue (H&E stain, ×100). **(A)** P16, diffusely and strongly positive; **(B)** CEA, positive; **(C)** ER, negative; **(D)** PR, negative; **(E)** PAX-8, negative; **(F)** WT-1, negative; **(G)** p53, wild-type pattern; **(H)** Ki-67 index, approximately 95%.

After preoperative discussion with the patient and her family, total hysterectomy, left salpingo-oophorectomy, omentectomy, and appendectomy were performed. Exploration revealed no gross lesions on the surfaces of the liver, spleen, stomach, bowel, omentum, or appendix, and no apparent lymphadenopathy in the pelvic or para-aortic regions. The postoperative pathology (**Figure 6**) showed a HPV-associated cervical adenocarcinoma, Silva pattern B, measuring 2.0×1.5×1.0 cm, with tumor infiltrating nearly two-thirds of the cervical canal and involving the endometrium. Lymphovascular space invasion (LVSI) was present. Mucinous tumors in bilateral ovaries were immunohistochemically consistent with metastasis from cervical adenocarcinoma. The endometrium showed cystic atrophy, and myometrial LVSI was observed. No carcinoma was identified in bilateral fallopian tubes, omentum, or appendix. All surgical margins (vaginal wall) were negative. Immunohistochemistry of cervical tissue (**Figure 7**): p16 (diffuse and strong positive), CEA (positive), ER (negative), PR (negative), PAX-8 (negative), WT-1 (negative), P53 (wild-type), Ki-67 index (approximately 95%), CK (positive), Vimentin (negative). Ovarian immunohistochemistry (**Figure 8**): p16 (diffuse and strong positive), CEA (positive), ER (negative), PR (negative), PAX-8 (negative), WT-1 (negative), P53 (wild-type), Ki-67 index (approximately 95%).

## 3 Discussion

In clinical practice, the primary task in the differential diagnosis of ovarian tumors is to determine whether they are primary or metastatic. Due to the morphological similarities between some metastatic ovarian tumors and primary ovarian mucinous borderline tumors or endometrioid tumors under light microscopy, the misdiagnosis rate remains high (7). These metastatic tumors often originate from the appendix, colorectum, biliary tract, or cervix. Among these, distinguishing ovarian metastasis from cervical adenocarcinoma is particularly challenging. On one hand, ovarian involvement by cervical adenocarcinoma is rare (8, 9). On the other hand, in early-stage disease, clinical manifestations are often atypical: patients may lack symptoms related to cervical cancer metastasis, have a normal-appearing cervix on examination, and even test negative for high-risk HPV. These factors can contribute to delayed diagnosis.

In this case, the initial diagnosis was also influenced by the aforementioned reasons, as well as the ultrasound findings suggesting a high probability of benign cervical lesions, which lowered the threshold for cervical biopsy. Preoperative imaging evaluation had limitations: ultrasonography misinterpreted the endocervical lesion as a polyp; computed tomography (CT) failed to identify the primary cervical lesion due to obscuration of pelvic details by a large ovarian mass. Furthermore, positron emission tomography–computed tomography (PET-CT) was not performed preoperatively, and systematic screening for common primary sites such as the breast and gastrointestinal tract was not conducted, which may have impacted the accurate determination of the tumor origin. Learning from this case, we propose that for patients presenting with bilateral ovarian solid/cystic-solid tumors, especially those accompanied by ascites, a high suspicion of metastatic tumors should be maintained even if the cervix appears normal and cytology is negative. Endocervical curettage (ECC) or magnetic resonance imaging (MRI) should be considered essential components of the preoperative evaluation. MRI, with its superior soft tissue resolution, may be more effective in detecting obscure cervical lesions.

Currently, a widely accepted approach to distinguishing primary from secondary ovarian tumors is based on tumor size and laterality: bilateral tumors of any size or unilateral tumors <10 cm in diameter are typically metastatic, whereas unilateral tumors ≥10 cm are mostly primary. This method can accurately classify the majority of tumors (10). Metastases mimicking primary ovarian tumors most commonly originate from the gastrointestinal tract, pancreas, biliary system, or cervix (primary cervical adenocarcinoma) (10, 11). Characteristic features of metastatic disease typically include bilateral ovarian involvement and a nodular growth pattern; however, they may also manifest as large unilateral masses exhibiting fused glandular architecture or expansile growth. In this case, the patient presented with massive ascites and bilateral ovarian masses, which should prioritize consideration of gynecologic malignancies, particularly metastasis from cervical adenocarcinoma.

To clarify the nature of the ovarian lesion, a series of immunohistochemical stains were performed. The results revealed an entirely consistent immunophenotype between the ovarian and cervical lesions: diffuse strong positivity for p16, negativity for ER and PR, negativity for PAX-8, negativity for WT-1, a wild-type p53 expression pattern, and a remarkably high Ki-67 proliferation index of approximately 95%. Immunohistochemistry aids in differential diagnosis, with diffuse strong positivity for p16 serving as a sensitive and specific marker for identifying HPV-associated cervical adenocarcinoma (12). First, primary ovarian mucinous tumors typically exhibit p16 negativity or focal positivity, along with a Ki-67 index usually below 30%. In contrast, the combination of diffuse strong p16 positivity and a remarkably high Ki-67 index (approximately 95%) in the present case strongly argues against this diagnosis. However, as emphasized by Alexandra et al., since p16 expression lacks absolute specificity, confirmation of cervical origin for ovarian metastases requires demonstration of either identical chromosomal HPV DNA integration sites or concordant HPV genotypes between ovarian tumors and primary cervical carcinomas (13).Since the cervical adenocarcinoma in this case was an incidental postoperative finding, direct HPV DNA PCR testing was not performed on the surgical specimen. However, according to the World Health Organization (WHO) classification criteria, diffuse and strong positivity for p16 in cervical adenocarcinoma is widely accepted as a reliable surrogate marker for high-risk HPV infection. Combined with the typical histological features and immunophenotype of HPVA in this case (p16++, ER-, PR-), the findings meet the diagnostic criteria for HPV-associated (usual-type) endocervical adenocarcinoma. Although HPV DNA testing of tumor tissue would provide the most direct evidence, in clinical practice, the aforementioned immunophenotypic profile is recognized by the WHO classification as sufficient to establish a diagnosis of HPVA. Second, primary ovarian endometrioid carcinoma typically expresses ER/PR (14) and is frequently positive for PAX-8 (15). In our case, the negativity for these markers effectively rules out this possibility. Furthermore, ovarian high-grade serous carcinoma characteristically expresses WT-1 and exhibits a mutant-type p53 pattern (16, 17); the findings of WT-1 negativity and a wild-type p53 pattern in our case are inconsistent with this diagnosis. While confirmatory tests such as CK7/CK20 or CDX2 were not performed to further exclude a metastatic carcinoma of gastrointestinal origin, the presence of a definitive cervical primary, coupled with diffuse strong p16 positivity—a highly indicative marker of HPV-associated malignancy—clearly points to a cervical origin. In summary, the immunophenotype of the ovarian lesions is inconsistent with all common types of primary ovarian tumors but is entirely congruent with that of the primary cervical focus. This supports the final diagnosis: cervical adenocarcinoma with bilateral ovarian metastases. A distinctive feature of this case was the intraoperative finding of abundant yellowish gelatinous ascites. The presentation of gelatinous ascites is exceedingly uncommon in metastatic cervical adenocarcinoma. The source of ascites was a key diagnostic concern. Pseudomyxoma peritonei (PMP), a rare clinical entity with an estimated incidence of 2 per 10,000 cases (18), is characterized by peritoneal, omental, or visceral surface implantation of mucin-secreting cells leading to massive accumulation of gelatinous mucinous ascites. Imaging characteristics typically include: (1) parenchymal scalloping deformities (hepatic/splenic surfaces), (2) stellate mesenteric infiltration, (3) parenchymal invasive lesions, and (4) peritoneal mucinous implants. Pathogenesis note: The disease predominantly originates from appendiceal neoplasms, while ovarian lesions are generally secondary (19). The co-occurrence of endocervical adenocarcinoma and PMP is an exceptionally rare clinical scenario. In 2008, Smith et al. first established the clinicopathological link between these entities through a case of PMP developing 8 years post-hysterectomy for cervical adenocarcinoma (20). More recently, Sarita et al. (2024) reported primary cervical adenocarcinoma with PMP, characterized by gelatinous ascites with bilateral ovarian metastases—features remarkably similar to those in the current case (21).

Similar to the current case, Sarita’s report also described gelatinous ascites with bilateral ovarian metastases. The authors hypothesize that cervical adenocarcinoma cells may spread via retrograde menstruation to the peritoneal cavity, exceptionally co-occurs with PMP-associated mucinous ascites, though the precise mechanism remains unclear. Notably, Sarita et al.’s case demonstrated a grossly identifiable cervical lesion requiring biopsy, making the detection of adenocarcinoma non-occult. In contrast, our case involved occult cervical adenocarcinoma. Additionally, the absence of omental metastases was observed in our patient. Integrating radiological and immunohistochemical findings, we propose a diagnosis of occult HPV-associated cervical adenocarcinoma mimicking pseudomyxoma peritonei. The copious, pale-yellow, gelatinous fluid observed intraoperatively likely originated from ovarian metastases secondary to cervical adenocarcinoma, rather than a true concurrent PMP. The underlying mechanism may relate to the tumor’s distinctive mucin-producing phenotype, though specific pathways warrant further investigation.

The ovarian metastasis rate of cervical adenocarcinoma is higher than that of squamous cell carcinoma, with an average incidence of 5.31% (22). Risk factors include initial FIGO stage, histological subtype, and depth of stromal invasion in the primary tumor (22, 23). Notably, Shimada et al. demonstrated no correlation between ovarian metastasis and lymph node involvement or parametrial invasion. The metastatic pathways remain controversial, with proposed mechanisms including: (1) lymphatic or hematogenous (vascular) spread; (2) retrograde uterine/tubal dissemination of neoplastic cells (24, 25).In this case, pathological evaluation revealed extensive lymphovascular space involvement, suggesting predominant lymphatic dissemination rather than hematogenous spread. Definitive identification of the metastatic route requires additional immunohistochemical analysis: lymphatic metastasis is typically associated with D2-40 (podoplanin) positivity, whereas hematogenous metastasis shows CD34 positivity. Furthermore, the potential for transtubal metastasis cannot be excluded: tumor cells may disseminate cervico-uterotubally to the ovarian surface, with subsequent rupture of metastatic foci potentially resulting in massive mucinous ascites.

Undoubtedly, this represents a case of incidentally detected HPV-associated invasive cervical adenocarcinoma (Stage IVB, FIGO 2018) following total hysterectomy (26), with complete excision of the uterus with metastasectomy achieving negative vaginal surgical margins. Radiologic reassessment of whole-abdomen CT demonstrated no evidence of lymphadenopathy in pelvic or para-aortic regions. According to the 2025 NCCN Guidelines, we recommend definitive cisplatin-based chemoradiation without adjuvant surgical intervention. At the 3-month postoperative follow-up, the patient had completed the initial cycle of chemotherapy with no radiological evidence of recurrence; however, the long-term prognosis remains to be fully determined. This study will continue to monitor treatment efficacy, and updated prognostic data will be provided in the future.

## 4 Conclusion

In summary, the diagnostic approach to ovarian mucinous neoplasms requires rigorous exclusion of metastatic disease before considering primary ovarian origin. Secondary involvement by carcinomas from the appendix, colorectum, pancreas, biliary tract, or cervix is frequently misclassified as primary mucinous or endometrioid neoplasms per 2023 WHO criteria. Additionally, the occult metastatic cervical adenocarcinoma in this case presented with features mimicking pseudomyxoma peritonei, which is a rare occurrence. Therefore, this case highlights the necessity of integrating clinical presentation, pathological features, and imaging findings to identify metastatic cervical adenocarcinoma presenting as a mucinous ovarian tumor, particularly when the ascitic fluid character closely resembles pseudomyxoma peritonei.

## Data Availability

The original contributions presented in the study are included in the article/supplementary material. Further inquiries can be directed to the corresponding author/s.
